# Successful prophylaxis of ESBL Enterobacteriaceae repetitive urinary tract infections with subcutaneous temocillin: a case report

**DOI:** 10.1093/jacamr/dlad154

**Published:** 2024-01-05

**Authors:** Eloïse Delpierre, Jean-Philippe Lanoix

**Affiliations:** Infectious Disease Department, Amiens-Picardie University Hospital, FR-80000, Amiens, France; Infectious Disease Department, Amiens-Picardie University Hospital, FR-80000, Amiens, France; AGIR EA4294, Medicine Department, Université de Picardie Jules Verne, Amiens, France

## Abstract

**Objectives:**

Temocillin is an antibiotic belonging to the β-lactam family, introduced in 1988 but soon forgotten because of its narrow spectrum. Recently, it has been repurposed for its effectiveness against ESBL Enterobacteriaceae, and represents an alternative of choice to carbapenems due to its limited impact on the microbiota.

**Patient:**

We present here a successful case of antibiotic prophylaxis of recurrent ESBL urinary tract infections with subcutaneously administered temocillin.

**Conclusions:**

Temocillin is rarely administered subcutaneously and even more rarely in prophylactic situations. However, its tolerance profile and low impact on the microbiota should help reconsideration of its use in particular cases like this one.

## Introduction

The WHO considers antimicrobial resistance as ‘one of the biggest threats to global health, food security, and development’ and treats it as a global public health and socioeconomic priority.^1^ Indeed, bacterial antimicrobial resistance alone causes an estimated 1.27 million global deaths per year and 141 000 are caused by third-generation cephalosporin-resistant bacteria.^2^ Although France is not the most impacted by ESBLs in enterobacteria, this specific resistance represents 6.7% of urine samples and up to 19.5% for *Klebsiella pneumoniae*,^3^ leading to potential dramatic limitations of therapeutic options.

Temocillin, first introduced in 1988 in Belgium for the treatment of acute pyelonephritis, belongs to the β-lactam family, specifically penicillin. It is a bactericidal and time-dependent antibiotic, usable via IV and intramuscular routes only. Temocillin was later marketed for the treatment of lung infections caused by *Burkholderia cepacia* in patients with cystic fibrosis,^4^ and then for lower or upper urinary tract infections, bacteraemia and lower respiratory infections.^5^ This molecule was forgotten for a long time due to its narrow spectrum against Gram-negative bacteria and inactivity against anaerobes and *Pseudomonas aeruginosa*.^5–8^ However, given its activity against most ESBL-producing enterobacteria, the use of temocillin rose again. Furthermore, temocillin has the advantage of having a low impact on the microbiota, limiting the emergence of antibiotic resistance.^5,6,8–10^

Temocillin is currently recommended in the following indications: acute pyelonephritis caused by ESBL-producing bacteria after confirmation of sensitivity on drug susceptibility testing (temocillin should not be used empirically); male urinary tract infections if no other possible alternative is available (by French guidelines^5,11^); and bacteraemia associated with urinary tract infection (by European guidelines).^12^ However, temocillin is not recommended in the treatment of ESBL infection in the USA (only for the treatment of pulmonary infections caused by *B. cepacia* (IDSA and FDA recommendations).^13,14^

Whereas curative use of temocillin in ESBL infections and as a carbapenem-sparing strategy is validated and well evidence-based,^13–15^ its prophylactic use is not. We report a case of a patient suffering from recurrent severe and non-severe urinary tract infections caused by ESBL-producing bacteria. He was treated with subcutaneous temocillin prophylaxis for over 1 year, leading to a drastic reduction in ESBL infections.

## Case presentation

**Figure 1.**
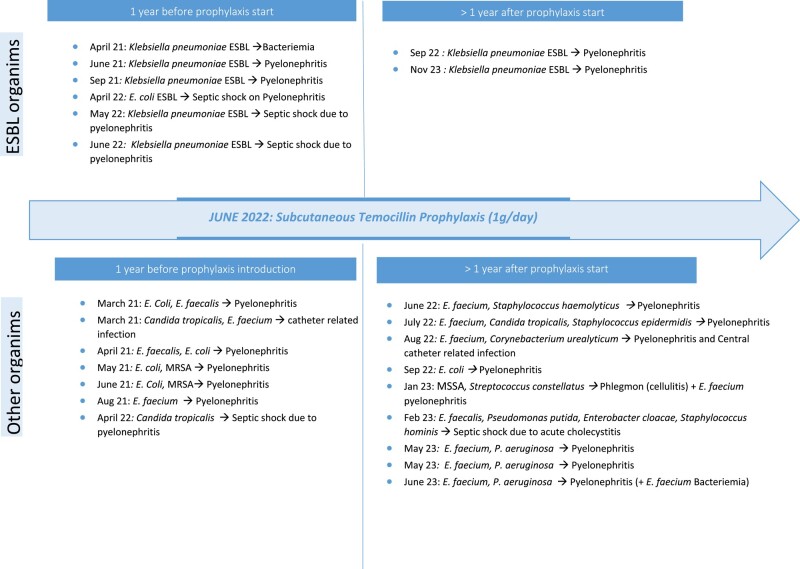
Two years of infections in our 51-year-old patient. The upper part lists infections with ESBL-producing organisms; the lower part lists those with non-ESBL-producing organisms. The left part lists infections 1 year before the introduction of temocillin; the right part lists those 1 year after the introduction of temocillin.

The patient was 51 years old and male, with history of right nephrectomy due to multiple coralliform lithiasis following childhood junction syndrome, non-insulin-dependent type 2 diabetes, hypertension, dyslipidaemia and multiple severe and non-severe urinary infections requiring recurrent hospitalizations and broad-spectrum antibiotic therapy (Figure 1).

Consequently, the patient needed a left nephrostomy tube in the context of obstructive lithiasic pyelonephritis on a solitary kidney. The patient also underwent cystoprostatectomy for neurogenic bladder. Nonetheless, he had no renal or hepatic insufficiency and had a normal nutritional status. He was admitted three times for septic shock due to ESBL-producing *K. pneumoniae* or *Escherichia coli* pyelonephritis. Additionally, the patient was hospitalized four times in the infectious diseases department for ESBL urinary infections. The patient received broad-spectrum antibiotics (piperacillin/tazobactam, imipenem, linezolid or ceftazidime) before and after the initiation of temocillin. The repetitive use of carbapenems before the introduction of temocillin did not prevent ESBL infections. All seven infections occurred over a 1 year period before we decided to start long-term suppressive therapy with temocillin 1 g once a day via the subcutaneous route.

Temocillin was administered subcutaneously (diluted in 100 mL of 0.9% saline) to avoid catheter-related infections, which were very common in this particular patient. Subcutaneous injections were administered by a home nurse following standard aseptic procedures. The 1 g per day dosing resulted in residual concentrations ranging from 9.5 to 16 mg/L, whereas, for curative usage, the residual expected concentration is usually above 12 mg/L.^16^

After 1 year of suppressive therapy, we observed a significant reduction in recurrent ESBL urinary infections while the patient kept having non-temocillin-susceptible infections (Figure 1). He had only one episode of septic shock over that year with a non-ESBL organism. Finally, he experienced neither local (injection site) nor systemic reactions. Extended analysis to find innate immunosuppression was performed, but did not reveal any. There was no change in infection control policy over the 2 years of follow-up (i.e. contact precautions and isolation at each hospitalization).

## Discussion

Repeated complicated urinary tract infections expose patients to repeated broad-spectrum antibiotic therapies, promoting the emergence of antibiotic resistance. Multiple studies have demonstrated the efficacy of temocillin against ESBL-producing Enterobacteriaceae infections;^7,10,13,14^ however, no studies have evaluated its suppressive subcutaneous use. Matzneller *et al.*^17^ compared IV and subcutaneous administration of 2 g dosing in eight healthy volunteers. They showed similar pharmacokinetic parameters with similar serum concentrations but slightly decreased overall exposure compared with IV administration. This suggested the equivalent efficacy of both routes. However, no studies have studied clinical efficacy or suppressive efficacy of temocillin subcutaneous administration, while pharmacokinetics and pharmacodynamics of subcutaneous administration of several antibiotics (e.g. ceftazidime, ceftriaxone and ertapenem) have been evaluated in suppressive therapy for bone and joint infection.^18^

Different residual plasma concentration assays yielded similar residual concentrations to that of curative dosing, as previously suggested.^12,17^ Given the numerous advantages of subcutaneous administration for patient comfort and prevention of bloodstream infections, we opted for its use in a patient with precarious venous access.

After 1.5 years of use, the patient did not present any infection with temocillin-resistant organisms. The frequency of resistant mutants *in vitro* ranges from 10^−8^ to 10^−10^ and mostly class B and class D β-lactamases are known to hydrolyse temocillin,^19^ although efflux mechanisms can cause high-level resistance.^19^ His remining infections were with temocillin-non-susceptible organisms, raising the question of Gram-positive prophylaxis.

### Conclusions

Although this is a single observation, we are convinced that in our global context of a high level of resistance and especially ESBL-producing enterobacteria, subcutaneous temocillin has a role in preventing severe infections and protecting particular patients from broad-spectrum antibiotics and acquisition of even more complex and dangerous MDR bacteria.
